# Supplementary figures and images for: Comprehensive analysis of CMTM family and immune infiltration in esophageal carcinoma
Source: PLoS One. 2025 Apr 3;20(4):e0321037. doi: 10.1371/journal.pone.0321037 (PMC11967974; doi:10.1371/journal.pone.0321037)

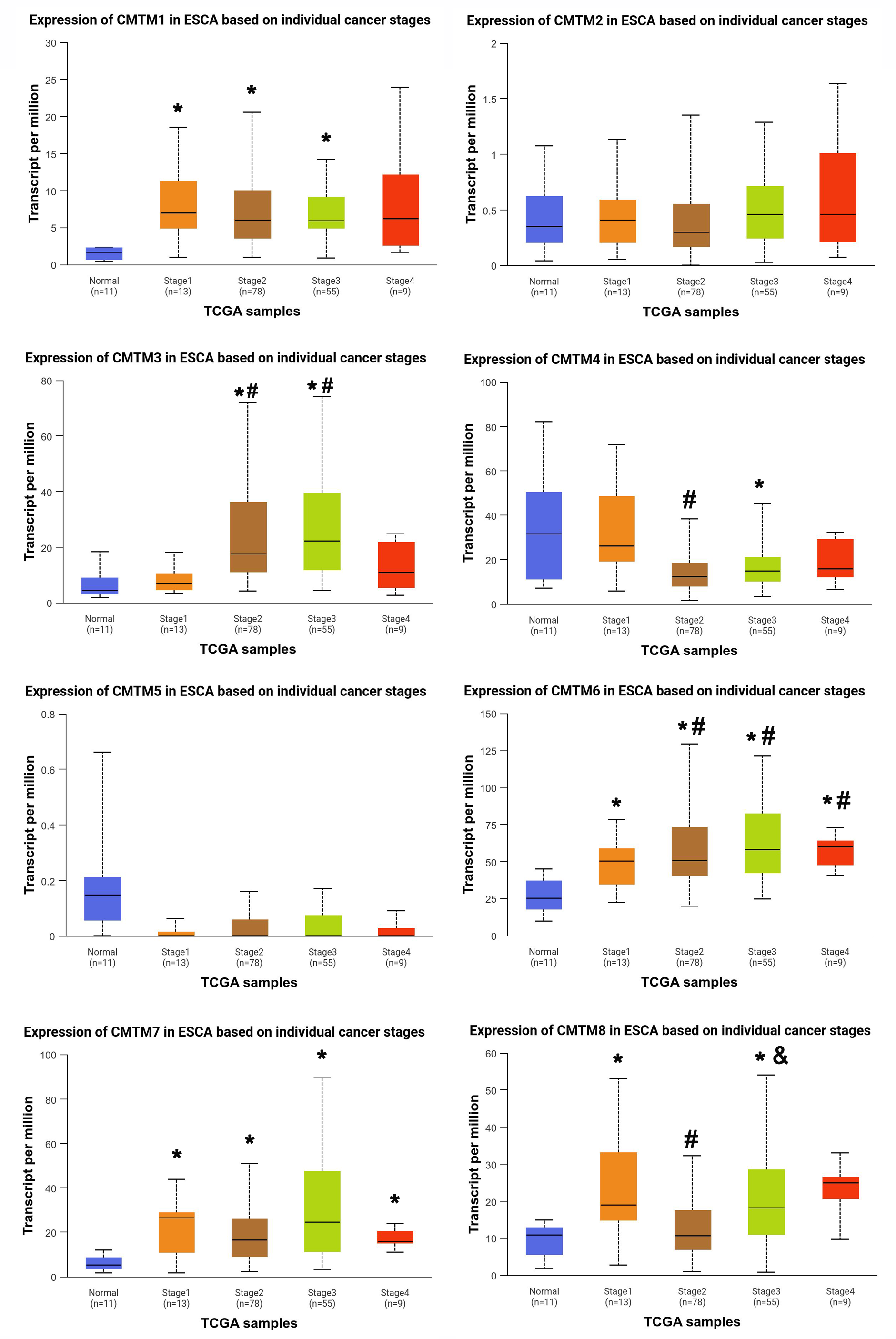

Supplement: S1 Fig — * P < 0.05, compared with normal tissue; # P < 0.05, compared with stage I; & P < 0.05, compared with stage II. (TIF) [file pone.0321037.s001.tif]
