# Supplementary material for: Comprehensive analysis of CMTM family and immune infiltration in esophageal carcinoma
Source: PLoS One. 2025 Apr 3;20(4):e0321037. doi: 10.1371/journal.pone.0321037 (PMC11967974; doi:10.1371/journal.pone.0321037)
Supplement: S1 Table — (DOCX) [file pone.0321037.s002.docx]

S1 Table Associations of clinical characteristics and CMTMs with mortality risk in ESCA patients

| Dependent | Alive (N=97) | Dead (N=60) | OR (univariable) | OR (multivariable) |
| --- | --- | --- | --- | --- |
| Gender |  |  |  |  |
| female | 19 (19.6%) | 4 (6.7%) |  |  |
| male | 78 (80.4%) | 56 (93.3%) | 3.41 (1.10-10.57, p=.034) | 3.83 (1.02-14.38, p=.047) |
| Age* | 61.8 ± 12.0 | 63.2 ± 11.6 | 1.01 (0.98-1.04, p=.460) |  |
| Stage |  |  |  |  |
| I | 14 (14.4%) | 3 (5%) |  |  |
| II | 53 (54.6%) | 23 (38.3%) | 2.03 (0.53-7.73, p=.302) | 1.26 (0.28-5.67, p=.760) |
| III | 29 (29.9%) | 26 (43.3%) | 4.18 (1.08-16.21, p=.038) | 1.27 (0.21-7.64, p=.798) |
| IV | 1 (1%) | 8 (13.3%) | 37.33 (3.31-421.57, p=.003) | 13.73 (0.94-199.76, p=.055) |
| T_Stage |  |  |  |  |
| T1 | 16 (16.5%) | 10 (16.7%) |  |  |
| T2 | 26 (26.8%) | 15 (25%) | 0.92 (0.33-2.54, p=.877) |  |
| T3 | 53 (54.6%) | 32 (53.3%) | 0.97 (0.39-2.38, p=.940) |  |
| T4 | 2 (2.1%) | 3 (5%) | 2.40 (0.34-16.97, p=.380) |  |
| N_Stage |  |  |  |  |
| N0 | 55 (56.7%) | 19 (31.7%) |  |  |
| N1 | 32 (33%) | 31 (51.7%) | 2.80 (1.37-5.75, p=.005) | 2.07 (0.73-5.83, p=.169) |
| N2 | 4 (4.1%) | 6 (10%) | 4.34 (1.11-17.06, p=.035) | 4.93 (0.78-31.05, p=.089) |
| N3 | 4 (4.1%) | 4 (6.7%) | 2.89 (0.66-12.73, p=.160) | 1.75 (0.28-10.95, p=.550) |
| NX | 2 (2.1%) | 0 (0%) | 0.00 (0.00-Inf, p=.989) | 0.00 (0.00-Inf, p=.989) |
| M_Stage |  |  |  |  |
| M0 | 87 (89.7%) | 46 (76.7%) |  |  |
| M1 | 1 (1%) | 3 (5%) | 5.67 (0.57-56.10, p=.138) |  |
| M1a | 0 (0%) | 5 (8.3%) | 29601486.71 (0.00-Inf, p=.987) |  |
| MX | 9 (9.3%) | 6 (10%) | 1.26 (0.42-3.76, p=.678) |  |
| CMTM family members* | |  |  |  |
| CMTM1 | 1.3 ± 1.0 | 1.2 ± 0.7 | 0.82 (0.56-1.21, p=.323) |  |
| CMTM2 | 0.3 ± 0.3 | 0.5 ± 0.4 | 3.17 (1.12-8.98, p=.030) | 2.76 (0.76-10.07, p=.123) |
| CMTM3 | 8.9 ± 7.6 | 7.8 ± 7.9 | 0.98 (0.94-1.03, p=.413) |  |
| CMTM4 | 7.8 ± 5.5 | 8.7 ± 7.4 | 1.02 (0.97-1.08, p=.380) |  |
| CMTM5 | 0.0 ± 0.0 | 0.0 ± 0.0 | 0.00 (0.00-257.24, p=.272) |  |
| CMTM6 | 24.2 ± 11.1 | 24.9 ± 9.6 | 1.01 (0.98-1.04, p=.688) |  |
| CMTM7 | 5.2 ± 4.6 | 5.3 ± 4.4 | 1.00 (0.93-1.08, p=.933) |  |
| CMTM8 | 8.8 ± 6.6 | 11.9 ± 9.0 | 1.06 (1.01-1.10, p=.019) | 1.01 (0.96-1.07, p=.635) |

*: Mean ± SD
