# Supplementary material for: Comprehensive analysis of CMTM family and immune infiltration in esophageal carcinoma
Source: PLoS One. 2025 Apr 3;20(4):e0321037. doi: 10.1371/journal.pone.0321037 (PMC11967974; doi:10.1371/journal.pone.0321037)
Supplement: Attachment — Submitted filename: Response to Reviewers.docx [file pone.0321037.s004.docx]

Dear Editors and Reviewers,

We sincerely thank the reviewers for their constructive and insightful comments, which have significantly improved the quality of our manuscript. We have carefully addressed all the concerns raised and made the necessary revisions accordingly. We believe that the revised manuscript is now more comprehensive and robust, and we hope that it meets the journal's standards for publication. Thank you once again for your valuable feedback and support.

Below, please find the comment in black, followed by our responses in red. Exact changes in the manuscript are also presented in red font.

**Responds to the reviewer’s comments:**

**Reviewer #1:**

1."CMTM3,4,8 expression was associated with the tumor stage of ESCA patients" While the p-values indicate that the differences shown in Fig. 1B is significant, the differences in the violin plot does not seem to be too noticeable for certain classes. For example, CMTM3 and CMTM4 on tumor stages 2 and 3, CMTM stages on stages 1 and 3. I think deeper analysis would be necessary. What patterns can be explained?

Thank you very much for your good advice, and it would be helpful to improve the manuscript. Fig. 1B was downloaded from GEPIA database (http://gepia.cancer-pku.cn/). The original data was unable to obtain to carry out deeper analysis. However, based on the TCGA database, the differences exist between tumor stages. Therefore, we added the deeper analysis at [Section Results 3.1]: “Further analysis based on TCGA database showed that CMTM3 was higher expressed in stageⅡ, Ⅲ than in stage Ⅰ (*P*<0.05), while CMTM8 was higher expressed in stage Ⅲ than in stage Ⅱ (*P*<0.05, S1 Fig).” Supplementary Figure 1 was also submitted as supplementary materials.

2. For better readability, I suggest splitting the contents in the discussion section into several subsections, each titled with 1 specific finding.

We sincerely thank the reviewer for this insightful comment. The comment is helpful to improve the manuscript. For better readability, subtitle has been added in [Discussion section].

3. On section 3.3, linear correlation was analyzed between CMTM expressions with the abundance of immune infiltrating cells. Several findings piqued my interest. Did you further measure the pairwise correlation between the CMTMs?

We sincerely thank the reviewer for this insightful comment. In our initial analysis, we focused on the correlation between CMTM expressions and immune infiltrating cells, as this was the primary objective of our study. While we did not perform pairwise correlations among CMTMs, we agree that this analysis could provide valuable insights into their potential interactions or co-regulation. On the other hand, this question initially caught my interest as well. After reading almost all the previous literatures about CMTMs research, we found that the CMTMs family members located on different chromosomes and had the diverse structure and function. According to previous studies, CMTM1-8 has distinct effects on malignant tumors as oncogenes or tumor suppressors in many human cancers. The detailed content was summarized in our previous article “Li J, Wang X, Wang X, Liu Y, Zheng N, Xu P, Zhang X, Xue L. CMTM Family and Gastrointestinal Tract Cancers: A Comprehensive Review. Cancer Manag Res. 2022 Apr 26;14:1551-1563. doi: 10.2147/CMAR.S358963. PMID: 35502328; PMCID: PMC9056025.” Although the pairwise correlation between the CMTMs has not measured, we will pay more attention on the relationships between CMTM4, 6 and PD-L1 in the future studies. Meanwhile, we also have discussed the members with similar structural and functional features as comprehensively as possible in the [Discussion section].

**Reviewer #2:**

1. page 6 - final sentence doesn't need to say p<0.05 twice. Could 95% confidence intervals be added to the survival curves in the figures?

We sincerely thank the reviewer for pointing out these issues.

Regarding the repetition of p<0.05 in the final sentence on Page 6, we have revised the sentence to remove the redundancy. The updated sentence now reads: [P<0.05 was considered statistically significant.].

Regarding the addition of 95% confidence intervals (CIs) to the survival curves, we agree that this would enhance the clarity and robustness of our results. We have now added 95% CIs to the survival curves in [Figure 3]. The revised figure has been updated accordingly.

**Reviewer #3:**

1. The study investigates the CMTM family in esophageal carcinoma (ESCA), focusing on immune infiltration. However, similar analyses have been performed for other cancers. What novel insights specific to ESCA does this study provide compared to prior research?

Thank the reviewer very much. Similar analyses about immune infiltration or on the roles of CMTMs have been performed for other cancers. In this study, we investigated the expressions of CMTMs and the relationship between CMTMs and immune infiltration in ESCA. We focus on better understand the underlying values of CMTM family members as potential molecular therapeutic targets and biomarkers in ESCA through performing an integrated bioinformatics analysis. We found that the expression levels of some CMTM members were significantly related to immune infiltrating cells in the TME. Therefore, it is speculated that CMTMs may become a new immunotherapy target for ESCA. As the roles of CMTM family members in other cancers, we have provided a direction for future research about immunotherapy and biomarkers in ESCA.

2. The study uses TCGA and GTEx datasets, but there is no indication of validation on independent datasets or real-world clinical samples.

We sincerely thank the reviewer for this valuable comment. We acknowledge that validation on independent datasets or real-world clinical samples would further strengthen the translational relevance of our findings. However, as a purely bioinformatics-based study, our primary goal was to leverage publicly available high-quality datasets, such as TCGA and GTEx, to identify potential biomarkers, pathways, or therapeutic targets. These datasets are widely recognized for their robustness and have been extensively used in similar studies to generate hypotheses and guide further experimental validation. Meanwhile, we added the related references at [Method section]. We have also clarified that this is a common limitation in purely bioinformatics studies and does not diminish the value of our findings as a starting point for further investigation in [Discussion seciton]. Based on this study, we will focus on o	exploring the functional relevance of the findings through in vitro or in vivo experiments in the future.

3. The manuscript focuses on the general ESCA population without stratifying between squamous cell carcinoma (ESCC) and adenocarcinoma (EAC), which are biologically distinct.

We sincerely thank the reviewer for this insightful comment. We agree that esophageal squamous cell carcinoma (ESCC) and esophageal adenocarcinoma (EAC) are biologically distinct entities with different molecular profiles, clinical behaviors, and therapeutic responses. In the initial analysis, we focused on the general ESCA population to identify overarching patterns and biomarkers that could be relevant across subtypes. However, we acknowledge that stratifying the analysis by ESCC and EAC could provide more precise and clinically actionable insights.

To address this concern, we have performed additional analyses in the revised manuscript. The results of these subtype-specific analyses are presented in the [Results section] and summarized in [Figure 2].

4. The manuscript highlights M2 macrophages and Tregs but does not provide sufficient mechanistic insights into how the CMTM family directly influences these immune cell populations.

We sincerely thank the reviewer for this insightful comment. We agree that a deeper mechanistic understanding of how the CMTM family directly influences M2 macrophages and Tregs would significantly strengthen our study. In the current study, our primary focus was to establish the correlation between CMTM expression and the abundance of immune cell populations, including M2 macrophages and Tregs. While our findings suggest a potential regulatory role of CMTMs in these immune cells, we acknowledge that the exact mechanisms remain to be fully elucidated.

We have now expanded the Discussion section 4.5 to include a more detailed hypothesis on how CMTMs might mechanistically influence M2 macrophages and Tregs. Specifically, we propose that [the presence of CMTM6 and CD58 on tumor cells significantly affects T cell-tumor interactions and response to PD-L1-PD-1 blockade. Therefore, CMTM6 is a promising target for developing immunotherapy.]. Additionally, we plan to investigate these mechanisms in future studies. We believe these experiments will provide deeper insights into the direct regulatory effects of CMTMs on immune cell populations.

5. The study relies heavily on bioinformatics analyses of TCGA and GTEx data without including experimental validations.

We sincerely thank the reviewer for this valuable comment. We acknowledge that experimental validation is crucial to confirm the findings derived from bioinformatics analyses. In this study, our primary goal was to leverage the extensive and well-curated datasets from TCGA and GTEx to identify potential correlations and patterns involving CMTM family members in cancer and immune regulation. These analyses provide a strong foundation for hypothesis generation and future experimental studies.

However, we agree with the reviewer that experimental validation would significantly strengthen our conclusions. While we were unable to include experimental validation in the current study due to the scope of the initial study, we have now expanded the Discussion section to explicitly address this limitation and propose future experimental approaches. Specifically, we plan to validate key findings using cell culture, immunohistochemistry and animal models. In addition, we have included a statement in the Limitations of Discussion section to highlight the need for experimental validation in future work. We believe these additions provide a more balanced perspective and set the stage for further investigation. We thank the reviewer for raising this important point and hope that our response and proposed future directions address their concerns.

6. Correlations between CMTM expressions and immune infiltration are reported without controlling for potential confounders.

Thank you for your careful review and valuable comments regarding the correlation between CMTM expression and immune infiltration in our study. We acknowledge that controlling for confounders is crucial for robust statistical analysis, and we regret this oversight in our initial manuscript. Due to limitations in data availability, we were unable to control for all potential confounders in our initial analysis. Now, we have performed additional analyses for key clinical variables, such as age, gender, and tumor stage. Although the association between CMTM family proteins and mortality risk was not significant in the multivariable analysis, the univariable results suggest that CMTM proteins may still play a role in the progression of esophageal cancer.

Meanwhile, due to the lack of detailed immune infiltration data in the TCGA database, we analyzed the relationships between CMTM family members and immune infiltration using TIMER 2.0. Previous research has shown that CMTM family members (e.g., CMTM6) play important roles in regulating immune cells (e.g., T cells and macrophages) in the tumor microenvironment. For example, CMTM6 has been reported to be associated with PD-L1 expression, a key molecule in tumor immune evasion. We speculate that CMTM family members may influence the progression and prognosis of ESCA by modulating immune cell infiltration. Therefore, their potential role in esophageal cancer warrants further investigation. We hypothesize that the effect of CMTM family proteins on mortality risk may be indirect, possibly mediated through mechanisms such as modulation of the tumor microenvironment or immune response, which were not fully captured in the multivariable analysis. We thank the reviewer for this valuable comment and will incorporate these suggestions into our future research plans.

The results are presented in the revised manuscript [Results section 3.2 and S1 table]. We have also added a discussion of this limitation in the revised manuscript, emphasizing the need for future studies to incorporate more comprehensive adjustments for potential confounders. Despite this limitation, we believe our study provides valuable insights into the role of CMTM family members in esophageal cancer and their potential interaction with the tumor immune microenvironment.

7. More references on bioinformatics-based workflow studies should be added to attract a broader readership i.e., PMID: 34572330, PMID: 37523012.

We sincerely thank for this valuable suggestion. We agree that incorporating additional references to bioinformatics-based workflow studies will enhance the relevance and appeal of our manuscript to a broader audience. We have carefully reviewed and added the suggested references (PMID: 34572330 and PMID: 37523012) to the revised manuscript [References 12, 13].

8. The markers used to identify M2 macrophages and Tregs (e.g., CD163, MS4A4A, VSIG4) are well-established, but the rationale for selecting these specific markers over others is not explained.

We sincerely thank the reviewer for raising this important point regarding the rationale for selecting specific markers. In the revised manuscript, we have added a detailed explanation in the [Methods 2.3] to clarify our choice of markers (CD163, MS4A4A, and VSIG4). These markers were selected based on their well-documented specificity and reliability in distinguishing M2 macrophages in the literature, as well as their relevance to the biological context of our study. For example, CD163 is a scavenger receptor highly expressed on M2 macrophages and is widely used as a marker for their identification. Similarly, MS4A4A and VSIG4 have been consistently reported as robust markers for M2 macrophages in various studies. We have also included additional references to support our selection of these markers, ensuring a more comprehensive justification for their use.

9. The manuscript uses *P*<0.05 as the significance threshold but does not address adjustments for multiple comparisons in large-scale data analyses.

We sincerely thank the reviewer for their insightful comment regarding the need for adjustments for multiple comparisons in our large-scale data analyses. We acknowledge that conducting multiple hypothesis tests without appropriate correction can increase the likelihood of Type I errors (false positives). we have now applied the Benjamini-Hochberg procedure to control the False Discovery Rate (FDR) across our statistical tests. The adjusted p-values have been recalculated and incorporated into the revised manuscript [Statistical Analysis and Results]. All results, tables, and figures have been updated to reflect the corrected p-values. We have ensured that only statistically significant findings after adjustment are highlighted as significant in the revised manuscript. We believe this adjustment strengthens the rigor and reliability of our findings. Thank you again for highlighting this important issue, and we hope the revised analysis addresses your concerns satisfactorily.

10. While the manuscript suggests that CMTMs may be immunotherapy targets, it does not discuss how this can be translated into actionable therapies.

We sincerely thank the reviewer for this insightful comment. We agree that discussing the translational potential of CMTMs as immunotherapy targets is crucial for highlighting the clinical relevance of our findings. In the revised manuscript, we have expanded the discussion in the [Discussion 4.5] to address how CMTMs could be translated into actionable therapies. We have also cited recent studies that support the feasibility of these strategies, providing a stronger foundation for the translational potential of CMTMs. We believe these additions significantly strengthen the manuscript and better align it with the interests of both basic and clinical researchers.

11. Without validation in clinical samples or patient-derived models, the translational relevance of the findings remains speculative.

We sincerely thank the reviewer for this valuable comment. We fully acknowledge that validation in clinical samples or patient-derived models would further strengthen the translational relevance of our findings. However, as a purely bioinformatics-based study, our primary goal was to identify potential biomarkers, pathways, or therapeutic targets using publicly available datasets and computational approaches. These findings provide a strong foundation for future experimental validation and clinical translation.

We have emphasized in the [Discussion section] that our analysis is based on robust computational methods and large-scale datasets, which have been widely used in the field to generate hypotheses and guide experimental research. We have also explicitly stated in the [Limitations subsection] that the lack of experimental validation in clinical samples or patient-derived models is a limitation of our study. However, we have also clarified that this is a common limitation in purely bioinformatics studies and does not diminish the value of our findings as a starting point for further investigation. We have included references [References 12, 13] to other high-impact bioinformatics studies that have successfully identified biomarkers or therapeutic targets without initial experimental validation, highlighting the accepted role of computational studies in hypothesis generation.

The sentence of Conclusion is changed to “It is speculated that CMTMs may become a new immunotherapy target. Further research on epigenetic changes, post-translational protein modifications, and related signaling pathways for CMTMs are required in ESCA.”

12. The study associates higher M2 macrophage and Treg abundance with reduced survival. How do CMTMs compare with other prognostic markers in ESCA?

We appreciate the reviewer’s insightful question regarding the comparison of CMTM family members with other prognostic markers in ESCA. In esophageal cancer, several well-established prognostic markers are commonly used, such as PD-L1, TP53 mutations, and immune cell infiltration (e.g., CD8+ T cells, M2 macrophages, and Tregs). Our study demonstrates that CMTM family members, particularly CMTM4 and CMTM6, exhibit significant prognostic value by correlating with immune cell infiltration, especially M2 macrophages and Tregs. Compared to traditional markers like PD-L1, which primarily reflect immune checkpoint status, CMTMs provide additional insights into the tumor microenvironment (TME) by modulating immune cell recruitment and function. Unlike single-gene markers (e.g., TP53), CMTMs are involved in broader regulatory networks, influencing both tumor cells and immune cells. For example, CMTM6 has been shown to stabilize PD-L1, linking it directly to immune evasion mechanisms. Our data showed that increased CMTM6 expression positively correlates with M2 macrophages. While M2 macrophages and Tregs are known to correlate with poor prognosis in esophageal cancer, our study highlights that CMTMs are upstream regulators of these immune cell populations. In conclusion, CMTM family members not only provide prognostic value comparable to existing markers but also offer unique insights into the immune microenvironment of esophageal cancer. Their dual role in tumor biology and immune regulation makes them promising candidates for both prognostic and therapeutic applications.
