# Supplementary material for: Comprehensive analysis of CMTM family and immune infiltration in esophageal carcinoma
Source: PLoS One. 2025 Apr 3;20(4):e0321037. doi: 10.1371/journal.pone.0321037 (PMC11967974; doi:10.1371/journal.pone.0321037)
Supplement: Attachment — Submitted filename: Response_to_Reviewers_auresp_2.docx [file pone.0321037.s005.docx]

Dear Editor and Reviewer 2,

We sincerely thank Editors and the reviewers for the insightful comments, which have significantly improved the quality of our manuscript. We have carefully addressed the concerns raised and made the necessary revisions accordingly. We believe that the revised manuscript is now more comprehensive and robust, and we hope that it meets the journal's standards for publication. Thank you once again for your valuable feedback and support.

Below, please find the comment in black, followed by our responses in red. Exact changes in the manuscript are also presented in red font.

**Responds to the reviewer’s comments:**

**Reviewer #2:**

1. I just wish to confirm that all the p-values quoted in the text and in the figures were the FDR p values? Often the one on the figures are not, particularly if p-values from different types of analyses are included. Currently in the text it seems like they are - so just confirming that. Sometimes in studies they quote an uncorrected and then a corrected (or adjusted) p-value. Would that be appropriate here (maybe only for most significant results?) to see the impact of the FDR adjustment?

Thank you for your thoughtful comments and for highlighting the importance of clarifying the p-values used in our study. We appreciate your suggestion to ensure rigor in our statistical reporting. We used FDR correction to account for multiple testing, which is a standard approach in high-throughput data analysis to reduce the risk of Type I errors. For example, in the Results section, we now report: ESCA showed significantly higher expressions of CMTM1,3,6,7 and lower expressions of CMTM4,5 than normal tissue (*P*<0.05, FDR-adjusted).

We have also updated the figure legends to clearly indicate that the p-values shown are FDR-adjusted. For example: "Figure 2. The differential expressions of CMTMs between ESCC and EAC (*P*: FDR-adjusted)."

We believe these changes enhance the clarity of our statistical reporting. Thank you again for your valuable feedback, which has helped us improve the manuscript.
